# Image-Driven Modeling of Nanoscopic Cardiac Function: Where Have We Come From, and Where Are We Going?

**DOI:** 10.3389/fphys.2022.834211

**Published:** 2022-03-08

**Authors:** William E. Louch, Harmonie Perdreau-Dahl, Andrew G. Edwards

**Affiliations:** ^1^Institute for Experimental Medical Research, Oslo University Hospital and University of Oslo, Oslo, Norway; ^2^K.G. Jebsen Centre for Cardiac Research, University of Oslo, Oslo, Norway; ^3^Simula Research Laboratory, Lysaker, Norway

**Keywords:** excitation contraction coupling (ECC), ryanodine receptor (RyR), super resolution microscopy, calcium induced calcium release, mathematical modeling

## Abstract

Complementary developments in microscopy and mathematical modeling have been critical to our understanding of cardiac excitation–contraction coupling. Historically, limitations imposed by the spatial or temporal resolution of imaging methods have been addressed through careful mathematical interrogation. Similarly, limitations imposed by computational power have been addressed by imaging macroscopic function in large subcellular domains or in whole myocytes. As both imaging resolution and computational tractability have improved, the two approaches have nearly merged in terms of the scales that they can each be used to interrogate. With this review we will provide an overview of these advances and their contribution to understanding ventricular myocyte function, including exciting developments over the last decade. We specifically focus on experimental methods that have pushed back limits of either spatial or temporal resolution of nanoscale imaging (e.g., DNA-PAINT), or have permitted high resolution imaging on large cellular volumes (e.g., serial scanning electron microscopy). We also review the progression of computational approaches used to integrate and interrogate these new experimental data sources, and comment on near-term advances that may unify understanding of the underlying biology. Finally, we comment on several outstanding questions in cardiac physiology that stand to benefit from a concerted and complementary application of these new experimental and computational methods.

## The Complementary History of Imaging and Computation in Cardiac Excitation-Contraction Coupling

**FIGURE 1:**
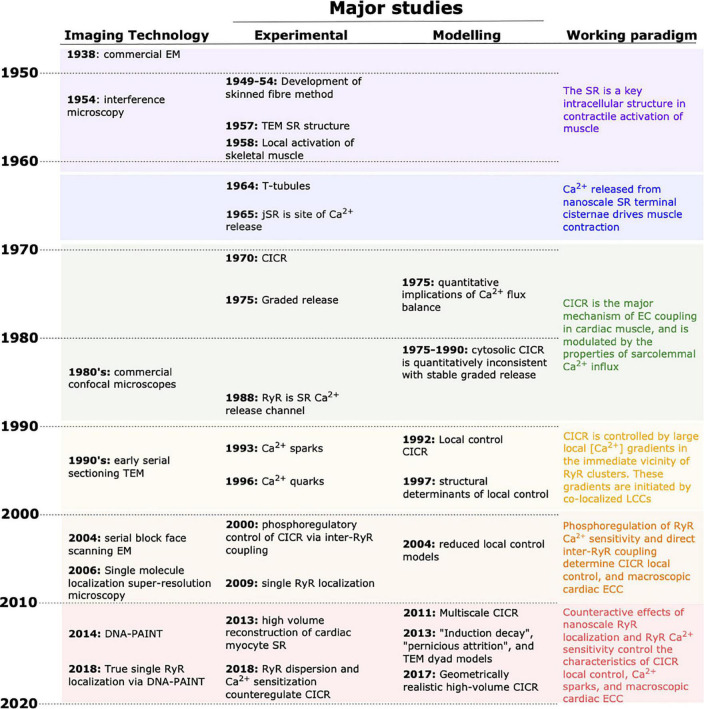
Progress in unraveling cardiac EC coupling based on experimental imaging and mathematical modeling. Continual advancement of imaging technologies **(left column)** has provided the tools for critical experimental insight during the last decades. Complementary advancements in mathematical modeling have contributed to shifting the paradigm in our understanding of EC coupling **(right column)**. In recent years, experimental and modeling developments have allowed a near merging of the spatial and temporal scales that each can be used to investigate. EM, electron microscopy; TEM, transmission EM; CICR, calcium-induced calcium release; SR, sarcoplasmic reticulum.

As in many fields of physiology, experimental and *in silico* approaches have each contributed elements critical to unraveling cardiac excitation–contraction coupling (ECC) during the past 60 years. However, it is only rarely that studies of both types have been designed in a coordinated manner, with one providing essential information to the other. Experimental approaches have always provided the foundation of the field, but as our understanding has developed it has repeatedly required investigation of structures and function that were unobservable via available methods. At those points, mathematical approaches have often been applied to crystalize the prevailing arguments and assumptions, and to provide clear, quantitative, and actionable postulates that have sometimes reoriented the field. These contributions of the subdisciplines are depicted in Figure 1, along with key (preceding) technological advances, and the resulting shifts in the working paradigm. Over the past decade, a number of major advances in microscopy have been applied to cardiac ECC, and provided a basis for entirely new approaches to coordinating experimental and computational studies. In this review, we describe key moments in the broader history of the discipline, and discuss how these new and burgeoning technologies may change the nature of investigation in the coming years. It is an exciting period for the field, as these new approaches promise to reconcile old controversies, and permit a degree of common understanding among modelers and experimentalists that has thus far largely been limited to the neurosciences.

### Structure as the Basis of Function

Transmission electron microscopy (TEM) provided the earliest foundation for our understanding of nanoscale ECC in both cardiac ventricle and skeletal muscle. These studies were among the first applications of electron microscopy in biology and part of extensive structural characterizations performed by Porter and Palade (1957). Their work established the existence and basic structure of the sarcoplasmic reticulum (SR) in striated muscle, and specifically identified the “diad” structures (“triad” in skeletal muscle) as sites of possible importance in ECC. These structures were formed by bulbous projections of the SR several hundred nanometers in diameter termed “terminal sacs” or “terminal cisternae,” which enwrapped a central tubular membrane that was later shown to be continuous with the sarcolemma (Franzini-Armstrong and Porter, 1964a,b). Together these elements provided a basis for sarcolemmal excitation to trigger Ca^2+^ release deep in the interior of the cell, thus verifying A F Huxley’s remarkable local stimulation experiments in skeletal muscle, which had pointed to their existence nearly a decade earlier (Huxley and Taylor, 1958). Oxalate and Ca^2+^ labeling of TEM sections established that the SR cisternae were indeed a major intracellular Ca^2+^ depot, at least in resting tissue (Huxley and Taylor, 1958; Constantin et al., 1965; Winegrad, 1965a). Winegrad’s (1965b) elegant follow-up study then put the final nail in the coffin by demonstrating transfer of Ca^2+^ from the cisternal structures to the myofilaments after K^+^-induced depolarization. At this point (the late 1960s), most aspects of the ECC mechanisms in cardiac and skeletal muscle remained to be established, but the fundamental membrane structures were more-or-less clear.

### The “Fuzzy” Foundation of Ca^2+^-Induced Ca^2+^ Release

Our basic concept of how these nanoscopic structures participated in cardiac ECC was introduced in the 1970s (Endo et al., 1970; Fabiato and Fabiato, 1972, 1975a,b,^1977^, ^1978^; Endo, 1977), well before modern nano-scale optical methods had been developed. As such, none of these early studies employed advanced imaging or computation, and many predated relatively straightforward measurement of intracellular Ca^2+^ via fluorescent dyes (Fabiato, 1981; Cobbold and Bourne, 1984). Still, through careful experimental design and interpretation, they laid out the framework of molecular events underlying cardiac ECC that endures today. It should be noted that, while the majority of these early findings hold for both ventricular and atrial myocardium, the ultrastructural differences between the chambers impart major differences in the subcellular dynamics of Ca^2+^-induced Ca^2+^ release (CICR). Unless otherwise noted, we focus on the nanoscale structure-function relationships in ventricular tissue throughout this manuscript.

By the early 1980s a number of characteristics of cardiac ECC function were clear. It was well known that the majority of Ca^2+^ that drives cardiac contraction is released from the cardiac SR at the terminal cisternae (now commonly known as the junctional SR, jSR). In intact myocytes, cardiac ECC clearly required Ca^2+^ influx during the action potential (Näbauer et al., 1989). However, the meticulous studies of Fabiato and Fabiato (1972, 1975b,c) showed that this could be recapitulated by rapidly increasing free [Ca^2+^] around the SR in skinned (sarcolemma removed) preparations (Szent-Gyorgyi, 1949; Endo, 2011). This CICR was subject to a number of modulators including cytosolic [Mg^2+^] and ATP, and importantly, it was proportional to the rate at which the “trigger” [Ca^2+^] was introduced to stimulate SR Ca^2+^ release. In the early to mid 1980’s the plant alkaloid ryanodine (a known inhibitor of SR Ca^2+^ release) was used to great effect for isolating, and reconstituting Ca^2+^ release function (Cannell et al., 1985; Fabiato, 1985; Fleischer et al., 1985; Maurer et al., 1985; Hymel et al., 1988). This established the ryanodine receptor (RyR) family as SR Ca^2+^ release channels, and recapitulated (at the single channel level) the important sensitivities of macroscopic CICR to cytosolic Ca^2+^, ATP, and Mg^2+^. Prior EM observations (in skeletal muscle) of these gigantic channels at the cytosolic face of the terminal cisternae suggested that they clustered together in an ensemble (Inui et al., 1987; Lai and Meissner, 1989), and most likely also operated in a coordinated fashion during SR Ca^2+^ release.

Together, this information suggested that CICR was the dominant mechanism of cardiac ECC, wherein Ca^2+^ influx via sarcolemmal L-type Ca^2+^ channels (LTCCs) during the action potential drives Ca^2+^ release from RyRs in the junctional SR. This conceptual model differed from skeletal muscle ECC, which occurred in very similar structures but was far less dependent on Ca^2+^ influx (Rios and Pizarro, 1988; Näbauer et al., 1989). Thus, at the beginning of the 1990’s a wealth of functional studies had established the basic macroscopic properties of CICR in cardiac tissue, and given rise to a series of hypothetical nanoscale mechanisms (Stern, 1992). However, due to shortcomings in functional imaging resolution, these mechanisms could not be directly probed at the time, and it was mathematical modeling that made the first critical strides in interrogating these hypotheses.

### Early Quantitative Modeling and the Birth of Local Control

In the period leading up to the early 1990s, computational investigations focused on reconstituting observable macroscopic ECC by integrating known characteristics of cardiac membrane ultrastructure with our understanding of Ca^2+^ transporter function. A number of early models managed to recapitulate dynamic ECC properties driven by Ca^2+^ flux balance, most prominently force-frequency relationships and staircase phenomena (Bassingthwaighte et al., 1973; Kaufmann et al., 1974; Adler et al., 1985; Hilgemann and Noble, 1987). However, these models were necessarily simplified, and all involved Ca^2+^ release mechanisms that were not solely tied to intracellular Ca^2+^. The necessity for such phenomenological approaches was due to one major quantitative shortcoming of the central tenet of CICR—*when the Ca*^2+^ *released by the SR is assumed to contribute to the same pool as the trigger Ca*^2+^, *SR release becomes a fully regenerative “all-or-none” phenomenon.* This was in stark contrast to Fabiato’s (and others’) clear observations that SR Ca^2+^ release was a graded phenomenon that depended on both the magnitude and velocity of triggering Ca^2+^ influx (Fabiato and Fabiato, 1975a,^1977^). In 1992, Stern (1992) provided a critical theoretical study that crystalized this paradox and made crucial postulates for how nanoscale function and dynamics in CICR could overcome the issue. That paper was directive for the field and the formal birthplace of our ‘local control’ understanding of cardiac ECC.

In essence, local control quantitatively formalizes the concept that individual Ca^2+^ release sites must operate at least semi-independently, as they respond to their own local Ca^2+^ concentration. With this consideration, graded and stable SR Ca^2+^ release becomes an intrinsic property of the system across a much broader range of triggering fluxes and SR Ca^2+^ loads than if Ca^2+^ release is driven by the bulk cytosolic pool of Ca^2+^. As part of his treatise, Stern formulated several key characteristics required for a local control system to operate in this way: (1) the Ca^2+^ released by an RyR into its own local environment must diffuse away from the channel with approximately the same (or faster) kinetics than those with which the RyR channels close—a reasonable assertion based on available evidence, and one that has held the test of time; (2) when the number of RyRs at a release site is more than one (best estimates at the time suggested an average of ∼10), there must be a separate mechanism (e.g., RyR inactivation, or local jSR Ca^2+^ depletion) to terminate the Ca^2+^ release. If such a mechanism does not exist, the local [Ca^2+^] that drives channel opening remains high and the various RyRs “trade off” in their opening over time, resulting in “latched” release. In part because of these clear postulates, one major direction that both experimental and computational efforts have pursued in the decades since has been to more accurately quantify the number of RyRs that participate at a release site. As Stern demonstrated, the interaction between this number and the local SR Ca^2+^ dynamics critically determines several micro- and macroscopic properties of cardiac ECC, notably including the mechanism of local Ca^2+^ release termination. With these fundamental functional characteristics becoming more clear, the terminology used to describe the structures involved in CICR also became more precise. The collection of RyRs at a single jSR terminal (thought to operate as a single coordinated functional unit), together with that local jSR volume and its releasable Ca^2+^, became known as a Ca^2+^ release unit (CRU). Stern himself coined the term “couplon” to describe the CRU together with the local LTCCs that control Ca^2+^ release. Below we describe how super-resolution imaging has since added some subtlety to these definitions by identifying geometric subsets of RyRs within the CRU that likely behave semi-autonomously depending on conditions (e.g., SR load, RyR modulation). Such subsets are often termed “clusters.”

In 1992, the simulations that Stern (1992) had implemented involving stochastically operating collections of RyRs were at the limit of computational tractability, even without solving for local diffusion of Ca^2+^. However, it was clear that if local control was indeed the core mechanism of CICR, there were broad implications for how these nanoscopic local events were integrated over the whole cell, and how they both depended on and determined the macroscopic properties of cardiac ECC. With this seed, and parallel developments in both computing and imaging power, a second major direction for ensuing work has been to develop an integrated understanding of the interdependencies between locally controlled CICR and macroscopic cardiac ECC.

### Ca^2+^-Induced Ca^2+^ Release in the Age of Ca^2+^ Sparks and Quarks

By the early 1990s it was presumed that, given suitable optical conditions, local spontaneous Ca^2+^ release events (resulting from probabilistic opening of RyRs) should be observable in cardiac muscle. Interestingly, it was also at this time that the technology required to detect these elementary events had begun to become widely available. Laser scanning confocal microscopy (LSCM) critically enabled separation of in-focus and out-of-focus fluorescence, and built upon the basic concept for confocal optical sectioning developed and patented by Marvin Minsky in the late 1950s. At the time of Minsky’s patent, critical complementary technologies involving light collection, data processing and storage, and visualization were not yet available. Thus, it wasn’t until the late 1980s that commercial confocal microscopes began making their mark on biology, and could be applied to discerning these small localized Ca^2+^ release events. Only one year after Stern’s paper, Heping Cheng working with Jon Lederer and Mark Cannell, published the first measurements of these spontaneous Ca^2+^ release events that many had envisaged (Cheng et al., 1993). In that classic study, they utilized LSCM to visualize and characterize the events, naming them Ca^2+^ *sparks*, and the paradigm of CICR quickly shifted to focus on Ca^2+^ signaling in the dyadic nanodomain. It is particularly noteworthy that Cheng’s sparks exhibited a range of morphologies that closely reflected the various dynamic regimes predicted by Stern’s computations, including the non-terminating events reflecting “latched” release. Since 1993 many studies have been conducted to extend our understanding of spark dynamics. In particular, considerable efforts have been made to establish the similarities and differences in Ca^2+^ release dynamics for these spontaneous local sparks versus cell-wide CICR driven by the action potential and opening of LTCCs (“triggered” CICR).

A technical aspect of spark measurements that remains significant today, is that signal-to-noise characteristics for modern Ca^2+^ fluorophores in conventional LSCM imaging volumes are typically not high. As a result, discernible sparks are often only those events that involve a relatively large regenerative Ca^2+^ release centered within the imaging volume. Many smaller or out-of-focus events simply contribute to the background fluorescence and noise. Recognizing this, experimentalists have since sought to observe and characterize these smaller events, and modelers have focused on understanding their contribution to triggered CICR and importance to macroscopic ECC. Ca^2+^ signals resulting from the opening of single RyRs were first suggested by Peter Lipp and Ernst Niggli, and named quarks for their presumed quantal underpinnings (Lipp and Niggli, 1996; Niggli, 1999). While those clever studies required decidedly indirect approaches to measuring these events, more recent work involving simultaneous monitoring of cytosolic and SR Ca^2+^ has established that sub-spark events are both measurable and frequent in cardiac cells (Brochet et al., 2010). While it is unclear from this work if these events reflected the opening of single RyRs or small RyR clusters, it is noteworthy that these small release events are relatively heterogeneous, both in size and kinetics. This variability may reflect differences in both local Ca^2+^-mediated gating of RyRs and the geometric configuration of the RyR ensemble in the CRU.

In addition to an evolving understanding of RyR participation in Ca^2+^ sparks, experimental studies combining patch-clamp and confocal microscopy were providing new quantitative insight into triggered CICR. These studies reported the “gain” of this process in intact (non-permeabilized) myocytes, i.e., the quantitative relationship between LTCC-mediated sarcolemmal Ca^2+^ influx and RyR-mediated SR Ca^2+^ release, at the whole-cell level (Wier et al., 1994; Cannell et al., 1995). These studies brought forward a number of questions surrounding the efficiency of single LTCCs to trigger activation of the juxtaposed RyR ensemble—a property often described as ECC “fidelity.” However, due largely to inadequacies of LTCC antibody labeling, confocal imaging studies in this era struggled to identify the degree of LTCC and RyR colocalization, which limited the certainty with which these relationships could be established. A classic study from the Franzini-Armstrong group then managed to address this issue at far higher resolution. Using EM freeze fracture, they reported that RyR “feet” were indeed in close proximity to dimples in the membrane attributed to L-type Ca^2+^ channels (Sun et al., 1995). But how closely localized must these two channels be, and how many LTCCs must contribute Ca^2+^ to the dyadic cleft to enable high fidelity functional coupling? To investigate these fundamental geometric and quantitative requirements of CICR, Cannell and Soeller (1997) and Soeller and Cannell (1997) developed an idealized computational model of electrodiffusion in the dyad. They were particularly interested in understanding how very brief openings (mean open time ∼0.2 ms) of sarcolemmal LTCCs could trigger nearby RyRs (for which opening latencies are on the order of ∼1–2 ms). This required a comprehensive reaction-diffusion simulation framework and meticulous attention to the RyR dynamic model. Through these efforts, they established a number of principles that continue to guide CICR concepts and modeling today. First, the width of the dyad (the distance between the sarcolemmal and jSR surfaces) is critically important to the efficiency and fidelity of coupling. This is now a ubiquitous model finding, but one which had not been addressed in a comprehensive way by prior studies. Second, even within such a constrained space, the distance of individual RyRs from an open LTCC was crucial for determining their probability of opening, suggesting that LTCCs and RyRs must be tightly arranged in the dyad. It was also remarkable that, in the context of the very restricted dyadic space, opening of a single LTCC could dramatically increase the effective [Ca^2+^] in the immediate vicinity of adjacent RyR binding sites on the microsecond time-scale, thus allowing measured LTCC kinetics to reliably trigger its accompanying CRU. Having noted those key findings, Soeller and Cannell also readily acknowledged the lack of certainty surrounding aspects of RyR dynamics that made their precision somewhat more fortuitous than it may have appeared. Indeed, it is now clear that RyR gating kinetics, dyadic [Ca^2+^] sensitivity, and unitary current all importantly determine the ability for opening of either an LTCC or RyR to trigger regenerative release. Thus, there is a growing appreciation that *in vivo* coupling fidelity is a plastic property. Indeed, RyR dynamics and Ca^2+^ sensitivity are strongly sensitive to modulation by Mg^2+^, ATP, Calmodulin, and a host of post-translational modifications. RyR unitary current is also dynamic during the spark (due to jSR depletion), and important for determining the threshold for spark termination and inter-CRU CICR (Ca^2+^ waves) at high SR Ca^2+^ load (Guo et al., 2012). As these characteristics of single RyR function have become more clear (Fill and Copello, 2002; Guo et al., 2012), and SR Ca^2+^ dynamics somewhat more measurable (Zima et al., 2010), various models have been developed to fully capture both spark initiation and termination (Cannell et al., 2013; Gillespie and Fill, 2013; Laver et al., 2013). Virtually all of these studies have reinforced the roles of dyadic width, and radial position of RyRs in determining spark activation. All have also emphasized a critical role for the size of the depletable local SR Ca^2+^ pool (jSR Ca^2+^ content) for determining spark termination. These characteristics appear to be the most fundamental for enabling reproducible CICR resulting from LTCC or spontaneous RyR opening. However, a range of dynamic factors also exert important physiological modulation of CICR. For example, voltage-dependent enhancement of unitary LTCC current potentiates CICR (Cordeiro et al., 2004; Hund and Rudy, 2004; Harris et al., 2005). This is best established in the canine epicardium, where rapid early repolarization (prominent epicardial notch) increases unitary LTCC current and potentiates whole-cell CICR in a manner that is important for achieving transmurally synchronous contraction (Cordeiro et al., 2004; Hund and Rudy, 2004).

The determinants of spark characteristics described by those earlier studies have generally been supported by more recent experiments employing either simultaneous SR and cytosolic Ca^2+^ monitoring (Zima et al., 2008; Picht et al., 2011), or strong cytosolic buffering to constrain the released Ca^2+^ (yielding “Ca^2+^ spikes”) (Song et al., 1998). As a result, it has become clear that local Ca^2+^ release dynamics are quite different during triggered whole-cell CICR compared to spontaneous sparks. In turn, the roles of intra-SR Ca^2+^ diffusion and the local SR geometry have been brought into clearer focus as critical counterparts to RyR gating dynamics in determining the major aspects of sparks and macroscopic CICR. Significant modeling efforts have since been made to dissect the contribution of each to observable Ca^2+^ release dynamics. In particular, Hake et al. (2012), constructed a finite element model of a single jSR terminal (with associated regional network SR) from a 3D TEM tomogram to determine the role that geometry likely played in determining local SR Ca^2+^ depletion during a spark. An important immediate observation was that substantial Ca^2+^ must flow into the jSR from adjacent network SR regions to permit sparks of realistic duration and amplitude, even with considerable local calsequestrin buffering. Unfortunately, the scale of the dataset used to build the geometry was not sufficient to allow fully realistic intra-SR diffusion from outside of the imaged SR structures, and the speed of Ca^2+^ diffusion within the SR remains both contentious for experimentalists and a problematic uncertainty for modelers (Swietach et al., 2008; Picht et al., 2011). For that model, Hake et al. (2012) chose a simple and phenomenological model of RyR dynamics, in part to avoid another debated topic among experimentalists—the potential for luminal Ca^2+^ to promote RyR activation independent of cytosolic Ca^2+^. Several of the key modeling studies noted above found that such a mechanism was not necessary to observe major characteristics of a spark in reasonable geometries (for which jSR Ca^2+^ was depletable). However, a range of evidence had also suggested that luminal regulation is a feature of RyR function, and may be crucial for both normal and pathologic RyR behavior (Jiang et al., 2004, 2005; Priori and Chen, 2011; Tang et al., 2012; Chen et al., 2014; Peng et al., 2016). Most recently, Fill and Gillespie (2018) and Gillespie (2019) have provided an elegant pair of studies that appear to at least partially reconcile this argument. By analyzing the full closed and open time distributions (and correlations between paired events), they noticed that open time is both negatively related to the preceding closed time, and that the triple exponential open time distribution is qualitatively quite different to those of the 2-state RyR models used previously. By implementing these characteristics in a new model of RyR gating they observed that sparks spontaneously terminated at a critically low single RyR flux (and thus SR Ca^2+^ threshold), even when the SR is not depleted (Gillespie, 2019). This has provided an important basis for understanding many of the observations thought to support the role of SR Ca^2+^-sensing in RyR function and dysfunction, as well as a number of more general characteristics of RyR gating in CICR.

As a final note related to these studies of dyadic function, experimental data emerging in the 1990’s indicated that LTCCs need not be the sole triggers for RyR Ca^2+^ release. The Na^+^-Ca^2+^ exchanger (NCX), which predominantly functions in “forward” mode to extrude Ca^2+^ from the cell, reverses during the early repolarization of the action potential, due to the brief rise in cytosolic Na^+^ concentration and membrane depolarization. Various groups (Leblanc and Hume, 1990; Sipido et al., 1997) showed that Ca^2+^ influx by NCX can serve as at least a weak trigger for CICR. However, it was simultaneously noted that realistic unitary flux for reverse-mode I_NCX_ imposes a major quantitative constraint on the ability of intra-dyadic NCX to provide trigger Ca^2+^. Still, confocal imaging studies also appeared to confirm that a substantial proportion of NCX proteins were co-localized with RyRs, thus providing some structural support for this mechanism (Jayasinghe et al., 2009). To more comprehensively interrogate the quantitative constraints of this process, Lines et al. (2006) employed a mathematical model of the dyad accounting for diffusion of both Na^+^ and Ca^2+^. Their simulations indicated that, in addition to closely colocalized NCX and RyRs, CICR between the two proteins requires nearby placement of a Na^+^ channel and extremely slow Na^+^ diffusion. While ionic diffusion speeds within the dyad remain the subject of debate, this experimental and modeling work highlighted a growing appreciation for the complexity of the CICR process.

### A Fiery Start to Understanding Regulation of Ca^2+^-Induced Ca^2+^ Release

Given the clear importance of individual RyR gating dynamics and local interaction among RyRs, LTCCs, NCX, and SR Ca^2+^ load, it was natural for questions about control of those characteristics to take center stage for CICR research at the start of the new millennium. The particular topics that forged this new direction focused strongly on phosphoregulation of the RyR channel, and its implications for both physical and functional interaction of RyRs within the dyad. Those topics also became some of the most controversial in the field and stimulated heated debate that continues to smolder. While a proper treatment of this history is beyond the scope of this review, the most contentious aspects revolved around a line of work from the lab of Andrew Marks at Columbia. At the beginning of the 2000s, Marks’ group proposed that phosphorylation of RyR2 at Serine-2808 caused dissociation of the accessory protein, FK506-binding protein 12.6 (FKBP12.6) from the RyR ensemble, and that this in turn destabilized the RyR closed conformation causing more frequent unitary RyR openings (Marx et al., 2000; Gaburjakova et al., 2001; Huang et al., 2006; Lehnart et al., 2008). These findings constituted an exciting development because they implied that phosphoregulation of RyR may be critical to the acute cardioregulatory effects of β-adrenergic stimulation, particularly contributing to enhanced contractile function and arrhythmogenicity. No less, they provided a plausible and detailed mechanism, as RyRs were proposed to become hyperactive, even near resting cytosolic Ca^2+^ concentration, and thus “leaky.” However, along with those details, Marks had made the relatively bold claim that this particular phosphorylation may represent a nexus for a range of CICR dysfunction in disease, based largely on the observation that RyR was hyperphosphorylated at this site in heart failure. Together these claims triggered a vigorous response, and for the next decade (and more) most of the assertions in the original work have been subjected to intense scrutiny and counterarguments—see Eschenhagen’s excellent commentary for a comprehensive review (Eschenhagen, 2010). While the field has now mostly accepted that the original claim overemphasized the role of that particular phosphorylation site, and likely also FKBP12.6, other aspects of the original findings have stood the test of time. Perhaps most importantly, the boldness of the proposal and the resulting scrutiny have themselves served to considerably extend our knowledge of CICR and its regulation.

One of the most initially challenging concepts in Marks’ regulatory model was that a single phosphorylation could so markedly alter function of the giant RyR protein, and in turn lead to the myriad observed outcomes. While such a potent role for S2808 has since been largely rejected (Bers, 2012), clinically meaningful potency has been clearly established for the family of RyR point mutations underlying catecholaminergic polymorphic tachycardia (CPVT) (Jiang et al., 2004, 2005; Priori and Chen, 2011; Tang et al., 2012), and indeed for Ca^2+^/Calmodulin-dependent protein kinase II (CaMKII) phosphorylation of the nearby S2814 site. It is now broadly accepted that CaMKII phosphorylation at S2814 is capable of conferring the majority of outcomes originally claimed by Marks for protein kinase A-mediated phosphorylation of S2808 (Guo et al., 2006; Kohlhaas et al., 2006; Curran et al., 2010; Hashambhoy et al., 2010; Gonano et al., 2011). Specifically, S2814 is recognized to be hyperphosphorylated in many disease states, notably including heart failure, and is thought to contribute to disease etiology by increasing SR Ca^2+^ leak and promoting arrhythmia. Mice harboring phosphomimetic and phosphodeficient substitutions at S2814 recapitulate most of these outcomes (Oort et al., 2010). Finally, because CaMKII is a Ca^2+^ activated kinase, its activity is strongly evoked by the enhanced Ca^2+^ cycling elicited by β-adrenergic stimulation, and perhaps by Ca^2+^-independent interactions (Pereira et al., 2007, 2013; Ogrodnik and Niggli, 2010). For this reason, distinguishing the impacts of PKA versus CaMKII in the purely acute setting has largely been the domain of a somewhat blunt set of pharmacological tools.

Following these phosphoregulatory studies, a range of other post-translational modifications have been shown to alter RyR function and contribute to regulation of cardiac ECC. Earlier work focused on the role of disulfide oxidation (Terentyev et al., 2008; Belevych et al., 2009) and nitrosylation (Xu et al., 1998; Sun et al., 2008; Bellinger et al., 2009), both of which increase RyR open probability under conditions of oxidative stress, again notably during heart failure (Terentyev et al., 2008). The effects of disulfide oxidation appear to rely on enhanced luminal Ca^2+^ sensitivity (Terentyev et al., 2008) whereas S-nitrosylation may involve enhanced sensitivity to cytosolic Ca^2+^ (Sun et al., 2008). More recently, a line of studies from Ben Prosser and Jon Lederer have established a role for localized stretch-induced ROS signaling that modulates RyR activity on a beat-to-beat basis (Prosser et al., 2011, 2013a,b; Limbu et al., 2015). The contention of this work is that stretch-induced activation of NADPH oxidase in the vicinity of RyR permits ROS-mediated activation of RyR, and may define a physiologic role for RyR redox status in myocytes.

Beyond these regulatory impacts on single RyR function, it is also possible that the nanoscale organization of RyRs within the dyad is subject to acute regulatory signaling. Fu et al. (2016) have observed that RyRs phosphorylated at S2808 more readily colocalize with LTCCs, and in a manner that requires bridging integrator 1 (BIN1). This intriguing finding suggests that S2808-phosphorylated RyR molecules are recruited to the dyad; an observation that appears to be supported by higher resolution imaging studies that have tracked phosphorylated and dephosphorylated RyR (Sheard et al., 2019; Hurley et al., 2021).

In sum, the strong debates and vigorous effort that has ensued from Marks original studies has added a great deal to our understanding of regulatory influences in cardiac CICR. For the purposes of discussing how these regulatory effects impact structure-function relationships in CICR, it is true that the mechanisms generally converge upon either enhanced or reduced RyR Ca^2+^-sensitivity (observed as both cytosolic and luminal sensitivity). While the effects of regulation on other aspects of single RyR function need to be more comprehensively studied, this form of modulation has been a prominent finding in studies to date, and can be readily incorporated into modeling frameworks.

## The Changing Interaction of Experiment and Simulation in the Era of Single Molecule Imaging and Serial Sectioning Electron Microscopy

### Single Molecule Localization Microscopy and Our Evolving Understanding of CRU Structure and Function

**FIGURE 2:**
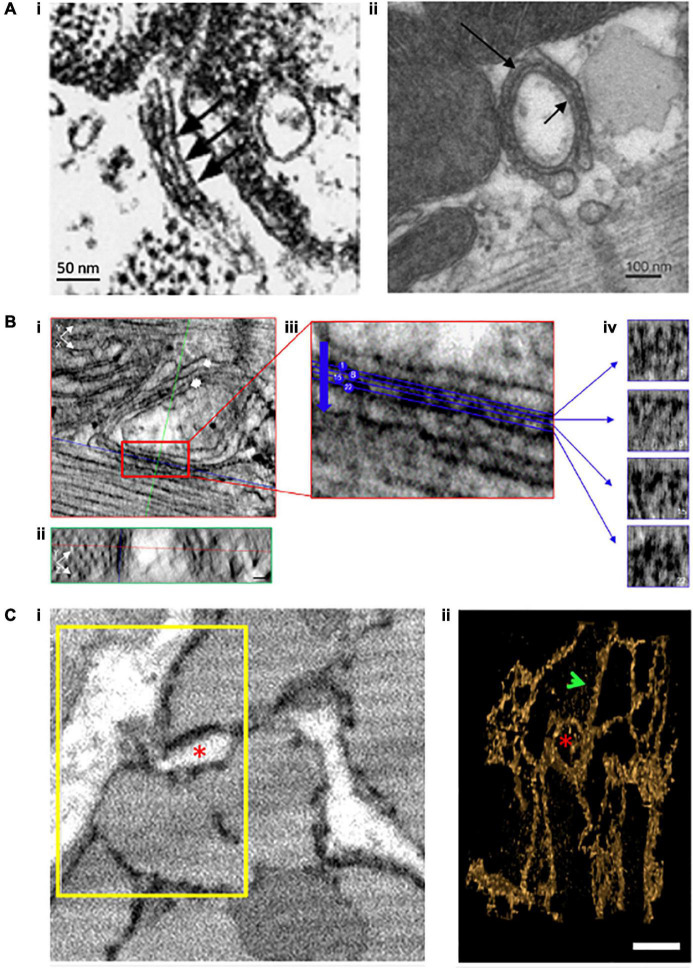
Evolving understanding of dyadic structure from electron microscopy (EM) studies. Visualization of dyads was first enabled by EM, as early as the 1960s. Even with 2D applications of this technique **(A)**, dyadic junctions between the jSR and the cell surface [**(i)**, chick myocardium, (Franzini-Armstrong et al., 1998)] or t-tubules [**(ii)**, rat ventricular myocyte, (Novotová et al., 2020)] were readily apparent, and highlighted the tight geometry of the dyadic cleft. Electron dense “feet” visible in the cleft (arrows) were attributed to the cytosolic portion of the RyR. With the advent of tomography techniques and enhanced contrast agents, it became possible to assess the 3D orientation of individual RyRs within dyads (**B**, scale bars = 30 nm, Asghari et al., 2020). XY **(i)** and YZ **(ii)** orthagonal views are presented for a representative dyad (single arrow = jSR, double arrow = t-tubule). Indicated planes are positioned on a single RyR (red line = XY, blue line = XZ, and green line = YZ plane). **(iii)** A magnified view of the boxed region in **ii** illustrates selected sections (4 of 28 illustrated) taken across the dyadic cleft. Each of these four sections is presented in **(iv)**, allowing identification of the position and orientation of individual RyRs. Full 3D rendering of cardiomyocytes has been made possible by serial block face imaging coupled with scanning EM (**C**, Colman et al., 2017). **(i)** The presented 2D image from a sheep cardiomyocyte illustrates dyadic junctions between t-tubules (white) and the electron dense SR (black). Reconstruction of the boxed region in 3D **(ii)** illustrates both network SR (arrow) and the jSR encircling a t-tubule (asterisk). Copyright permission was obtained for the reproduction of Panel **(Ai)** (Franzini-Armstrong et al., 1998). Copyright permission was not required to reproduce the other figures.

As noted in previous sections, it has long been realized that Ca^2+^ release occurs at discrete sites (i.e., CRUs), where cooperating RyRs generate Ca^2+^ sparks. Early estimates of CRU dimensions stemmed largely from 2D EM studies, using counts of RyR “feet” within the dyadic cleft (Figure 2A). These data were extended to 3D based on assumptions of dyadic shape, leading to the calculation that typical CRUs contained >100 RyRs, which were assumed to be densely packed in a single contiguous ensemble (Franzini-Armstrong et al., 1999). Data from studies employing confocal imaging coupled with deconvolution analysis appeared to support these estimates (Soeller et al., 2007), and led to the incorporation of such large, densely packed RyR arrays into mathematical models (Koh et al., 2006; Restrepo et al., 2008). Within these arrangements, RyRs were thought to be present in a “crystalline array,” based on data from both *in situ* and *in vitro* imaging showing RyRs oriented in a corner-to-corner, grid-like arrangement (Franzini-Armstrong and Protasi, 1997; Yin et al., 2005). However, emerging imaging techniques have now directly challenged these assumptions. In this section we highlight how these advances have triggered broad reconsideration of dyadic structure and function.

**FIGURE 3:**
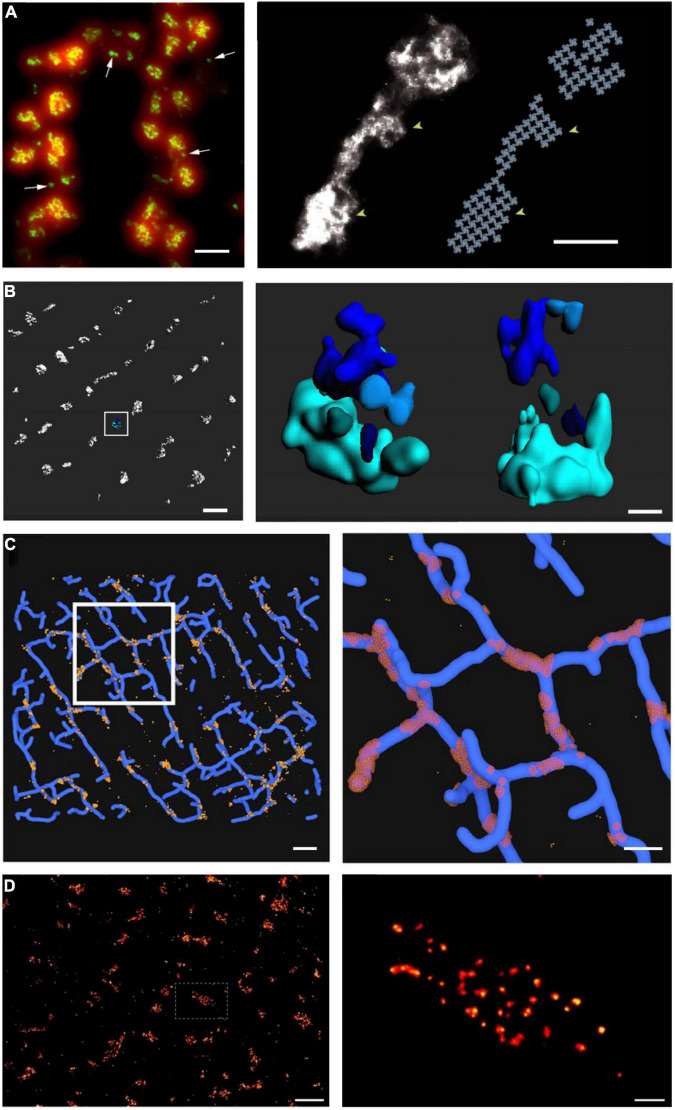
Advances in super-resolution imaging provide novel insight into RyR organization. Although previous work had suggested that RyRs are present in large clusters, dSTORM super-resolution imaging of RyRs has shown that the channels are rather present in multiple small, neighboring clusters. On the cell surface, these arrangements are present in double rows, on either side of z-lines [**(A, left panel)**, (Baddeley et al., 2009)]. The increased resolution of RyR imaging by dSTORM (green) is noted in comparison with diffraction-limited imaging (red). Maximal occupancy of imaged clusters was calculated based on an assumed grid-link arrangement of channels **(A, right panel)**. Quantitative assessment of more complex RyR arrangements within the cell interior requires high-resolution in 3D. Using 3D dSTORM, internal Ca^2+^ release units (CRUs) containing multiple RyR clusters with diverse configurations were observed [**(B)**, enlargement of indicated region at right; (Shen et al., 2019)]. To gain insight into the orientation of RyRs within dyads, correlative 3D dSTORM imaging of RyRs was combined with confocal t-tubule imaging, and the channels were localized at the interfaces of the two signals [**(C)**, enlargement of indicated region at right, (Shen et al., 2019)]. Even higher resolution imaging using the DNA-PAINT technique revealed that RyRs exhibit irregular packing within clusters [**(D)**, (Jayasinghe et al., 2018)], in contrast to grid-based assumptions. Scale bars in left, right panels: A = 500 nm, 100 nm; B = 500 nm, 100 nm; C = 1 μm, 500 nm; D = 1 μm, 100 nm. Copyright permission was not required to reproduce the figures. **(A)** Copyright 2009, National Academy of Sciences.

With the advent of super-resolution imaging (Betzig et al., 2006) came an unprecedented opportunity to investigate dyadic structure at the nanoscale. The two more commonly used techniques of single molecule light microscopy (SMLM) are photoactivatable and photoconvertible localization microscopy (PALM) and stochastic optical reconstruction microscopy (STORM) (Nieves et al., 2018). Both techniques employ the stochastic sampling of permanently bound fluorescent molecules switching between an on and off state, and subsequent reconstruction of an image with a spatial resolution on the order of 30 nm. Thus, these techniques offer an impressive (nearly 10-fold) improvement in resolution over traditional light microscopy techniques such as LSCM, and the ability to discern tiny dyadic components. To this end, the first cardiac application of SMLM was a dSTORM-based analysis of RyR organization within dyads at the cell surface of rat cardiomyocytes (Figure 3A; Baddeley et al., 2009). In contrast to the prevailing assumption at the time, these investigators reported that RyRs were not present in large ensembles, but rather in multiple small, neighboring clusters of a broad variety of shapes and sizes. Using a crystalline-array based filling of the identified cluster regions, Baddeley et al. (2009) calculated that the maximum occupancy of an average RyR cluster was only sufficient to hold approximately 14 channels, although many isolated single-channels were also observed. This insight required a major refinement of CICR models based on large (>100 RyRs), single-cluster CRUs. Xie et al. (2010) proposed that neighboring RyR clusters could coordinate the generation of a Ca^2+^ spark if their edge-to-edge distances were <100 nm. At these short distances, it was hypothesized that Ca^2+^ released from one cluster could jump to a nearby cluster, triggering its activation. Thus, according to this model, the basic unit of Ca^2+^ release in cardiomyocytes is a multi-cluster CRU or “super-cluster” of cooperating RyRs.

Of note, the seminal work of Baddeley et al. (2009) was conducted at the cell surface, where RyR arrangement was assumed to be roughly planar, enabling straight-forward estimation of channel numbers. The internal dyadic arrangements were expected to be far more complex, as the junctional SR curves around t-tubules (TTs) to form dyads, creating an arrangement where RyRs are super-imposed in the z axis. As with other optical imaging modalities, the resolution of dSTORM is considerably lower in the z axis than in the xy plane, meaning that it is not possible to discern vertically-aligned channels with 2D imaging. To circumvent this problem, Shen et al. (2019) employed phase-ramp dSTORM imaging to enable 3D imaging deep within rat cardiomyocytes (Figure 3B). In that study, we exploited an observed linear relationship between the number of fluorescent events (blinks) and the number of RyRs in images taken at the cell surface. Using this relationship for calibration, we estimated the numbers of RyRs within internal clusters and CRUs. These analyses showed that internal CRUs contain a highly variable number of rather small clusters (Figure 3B). While we found that many clusters contained only a single RyR, the average cluster (a fully contiguous RyR ensemble) contained 13 channels, and the average CRU (an RyR ensemble for which separation between individual channels did not exceed 100 nm) contained 23 channels (Shen et al., 2019). These estimates are significantly smaller than earlier estimates based on 2D EM (Franzini-Armstrong et al., 1999) or dSTORM of interior RyRs (Hou et al., 2015). However, they are in better alignment with EM tomography studies, which also further supported a complex, multi-cluster arrangement of internal CRUs (Hayashi et al., 2009). Of note, while the calibration-based approach to 3D imaging employed in Shen et al. (2019) enabled tallying of RyR numbers, it did not reveal the actual orientation of RyRs within clusters. Thus, correlative 3D dSTORM/confocal imaging of RyRs and t-tubules was employed to create an interface between the opposing jSR and TT membranes where the RyRs were arranged based on grid-based occupancy (Figure 3C). This analysis revealed that most RyRs are present in dyads in healthy cardiomyocytes, and that non-dyadic, “orphaned” or “rogue” RyRs are present in very small clusters. This latter observation supports that non-dyadic RyRs contribute to non-spark based “silent” RyR leak, since the released Ca^2+^ is of too low magnitude for detection by standard confocal techniques.

**FIGURE 4:**
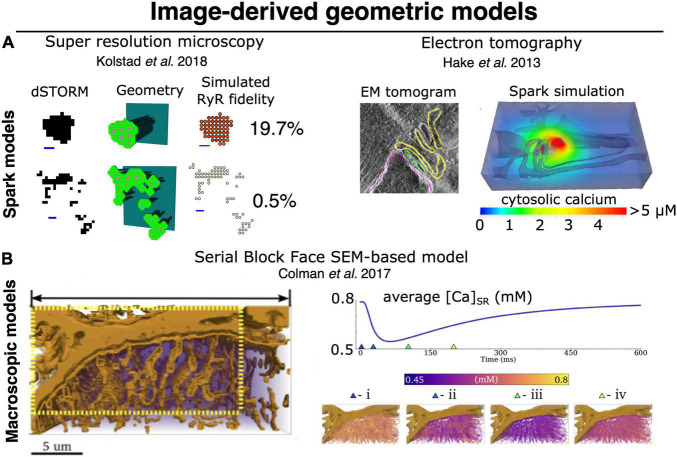
Image-derived approaches to geometric modeling of CRU and whole-cell cardiac ECC. **(A)** Image-driven models of single dyad (and CRU) structures have been implemented beginning with both super resolution light microscopy (dSTORM), (left) (Walker et al., 2014; Kolstad et al., 2018), and tilt-section tomography from high voltage TEM imaging (right) (Hake et al., 2012; Edwards et al., 2018). To date, no correlative light/EM datasets visualizing both protein and membrane localization have been employed for this purpose, although such approaches would be highly desirable. **(B)** High-volume EM methods, particularly SBF-SEM, have recently been used as a basis for constructing image-driven geometries for much larger subsections of a cardiac myocyte (Colman et al., 2017; Colman, 2019). Simulations in these geometries can be used to interrogate the interaction of local Ca^2+^ release with uncertain (and difficult-to-measure) physiological properties, such as intra-SR Ca^2+^ diffusion. At right, spatially distributed intra-SR Ca^2+^ concentration is shown at various times during the evoked macroscopic SR Ca^2+^ release. Models of this type will likely provide the basis for bridging between the stochastic dynamics of single CRUs (and single RyRs) and pseudo-continuous dynamics of macroscopic CICR, thereby allowing us to better deconvolve the multitude of factors known to influence both. Copyright permission was not required to reproduce the figures.

The novel, imaging-driven insights into CRU morphology described above have inspired new directions in mathematical modeling over the past decade. Based on the realization that RyR clusters are of irregular shapes, Walker et al. (2014) simulated the efficacy of Ca^2+^ spark generation with differing CRU configurations. They observed that more regular RyR cluster geometries exhibited higher spark fidelity than irregular shapes, in which individual RyRs have on average fewer neighboring channels. Cooperative “saltatory” Ca^2+^ release between clusters has also been studied in a number of mathematical models, based on both idealized (Nivala et al., 2012; Louch et al., 2013) and super-resolution defined CRU geometries (Figure 4; Macquaide et al., 2015; Kolstad et al., 2018). These analyses support the concept of super-cluster functionality; a paradigm that is somewhat analogous to the inter-CRU coordination that occurs at the micrometer scale during Ca^2+^ waves (Cheng et al., 1996; Keizer and Smith, 1998). These studies further indicated that the degree of cooperation between CRU sub-clusters also has significant implications for spark magnitude and kinetics (Louch et al., 2013; Macquaide et al., 2015; Kolstad et al., 2018). Of course, these aspects of CRU substructure interact strongly with RyR Ca^2+^ dependence and unitary current (as mentioned above) to determine local RyR interaction and release dynamics. The quantitative hierarchy of importance of structural versus RyR regulatory subtleties remains to be established, but it is clear that both are capable of markedly altering local control of CICR.

The dSTORM studies highlighted above estimated a maximum occupancy of each imaged RyR cluster based on a crystalline, lattice arrangement of the RyR channels reported in earlier work (Franzini-Armstrong and Protasi, 1997; Yin et al., 2005). However, with xy resolution very close to the actual size of the RyR (30 nm), it was not possible to assess intra-CRU packing in these dSTORM studies. Insight at that level (intra-CRU) has thus required the advancement of techniques with even higher resolution. Using tilt-EM tomography, Asghari et al. (2014, 2020) have shown that RyRs do not form an ordered pattern, but instead exhibit a more scattered arrangement even within clusters (Figure 2B). Similar results have been provided by a newer technique called DNA-PAINT. This approach was developed to achieve transient binding of fast-diffusing fluorescent DNA oligomers (“imager”) to a target of interest (“docker”) conjugated to antibodies (Sharonov and Hochstrasser, 2006; Jungmann et al., 2010). The repeated, transient binding events produce fluorescence blinking, allowing stochastic imaging at a resolution of <10 nm. Employing this technique, Jayasinghe et al. (2018) recently confirmed that RyR clusters exhibit irregular packing (Figure 3D). But what implications does this hold for function? Until recently, few modeling studies had interrogated the effects of RyR packing. Walker et al. (2014) observed that less dense RyR clusters exhibit lower Ca^2+^ spark fidelity, and Louch et al. (2013) reported that when sparks are successfully generated, the Ca^2+^ release is of reduced magnitude and slowed kinetics. Thus, by 2015, and thanks to the application of SMLM and structurally-defined modeling approaches, it was clear that CRU function (and even CRU definition) is critically dependent on the local arrangement of individual RyRs.

### Extending the Horizon: High Volume Membrane Mapping Through Serial Sectioning Scanning Electron Microscopy

While the development of SMLM has provided enormous power by mapping RyR cluster structures in large sections of a myocyte, these methods are not sufficient in-and-of-themselves to constrain models of structure-function relationships in CICR. This is because, as described above, RyR cluster size interacts with the local jSR Ca^2+^ pool to determine the dynamic regime of Ca^2+^ release. That is, the relationship between RyR cluster size and the local jSR size, and thus Ca^2+^ content, is critically important. When SMLM-defined RyR locations are used to build computational structures, one must make important assumptions about these relationships (Kolstad et al., 2018), none of which are well-constrained at this time. EM methods provide a means of imposing this constraint, because they can be used to map the jSR membrane structure, which yields the local depletable SR volume. However, until recently, technical challenges prevented this type of membrane mapping in volumes comparable to those now accessible via SMLM.

The EM studies described in earlier sections involved transmission EM performed on thin cryotome sections or freeze fractured surfaces (Sun et al., 1995; Franzini-Armstrong et al., 1999), occasionally with tilt tomography to assess 3D membrane structures within the section plane (Hayashi et al., 2009). While these methods provided the bulk of our early understanding of the cardiac membrane structures in cardiac ECC, they are also limited to assessing those structures in the relatively small volumes imposed by the thinness of the individual sections. Neuroscientists have had long-standing interest in mapping membrane structures in full 3D volumes to reconstruct neural microcircuits. Until the early 2000s, this had been achieved via the same thin-sectioning approach performed serially (and manually) through a tissue block. This was both extraordinarily laborious and challenging to achieve high-quality registration of the manually collected sections. Shortly into the new millennium, a new range of techniques began appearing involving a variety of automated approaches to serially section a fixed tissue block and then record a scanning EM image of the freshly exposed tissue plane. These sectioning approaches include automated serial block-face SEM (SBF-SEM) (Denk and Horstmann, 2004), focused ion-beam milling SEM (FIB-SEM) (Heymann et al., 2006), and automated tape-collecting ultramicrotomy SEM (ATUM-SEM) (Hayworth et al., 2006).

The unprecedented ability for these methods to map membrane structures at resolution of a few nanometers (zeptoliter voxels) (Pinali et al., 2013; Pinali and Kitmitto, 2014; Morgan et al., 2016), but in total tissue volumes approaching ∼1 mm^3^, have provided a new foundation for simulating multiscale dynamics in cardiac ECC. Importantly, the scale constraints applying to experiments of this type should not be underestimated. At this resolution and tissue volume, the duration of the sectioning and imaging alone is measured in weeks, and the final retrieved dataset approaches petabyte storage scales. Those challenges notwithstanding, the first study to apply these techniques in cardiac myocytes was performed by Pinali et al. (2013) and imaged voxels of ∼ 11 zL over 5–8 cells (∼150–240 pL) per tissue block via SBF-SEM. This study provided the first cell-wide mapping of cardiac SR structure, verifying it as a diffuse but entirely continuous depot for storage and transport of Ca^2+^ (Figure 2C). Their work additionally provided new understanding of the complexity of dyadic junctions between jSR terminals and an intricately branched t-tubule system. While these findings were not entirely unexpected, the depth of detail available via these datasets has provided the first basis for modeling and simulation adhering to full geometric realism of the intracellular Ca^2+^ handling machinery. The first follow-up simulation efforts have demonstrated both the power and challenges of applying data that span such a range of spatial scales. In order for simulations to remain tractable, Colman et al. (2017) were forced to construct their simulation geometry from a myocyte subsection (∼1/12th of the cell volume), considerably down-sample the model from the full imaging resolution, and apply homogeneous jSR sizes at all positions where the SR and TTs were observed to form dyads (Figure 4). In this way, the model likely captured dynamics that depend on the spatial distribution of the dyads within the cell, but failed to incorporate the nanoscale CRU dynamics described in the previous section, and which should (in principle) be accessible at the raw imaging resolution.

Nevertheless, with future improvements in computational power and efficient modeling approaches, high volume SEM imaging promises to provide a key platform for constraining our understanding of critical properties of cardiac ECC. In particular, simulations involving such realistic and large SR geometries could provide clear constraint on the rates of intra-SR Ca^2+^ diffusion and jSR Ca^2+^ refilling, which at this time remain poorly understood. This is noteworthy because these properties are central to aspects of macroscopic frequency dependence in cardiac ECC, and for the pathologic dynamics underlying Ca^2+^ waves, and electrical and mechanical alternans (Gaeta et al., 2009, 2010; Gaeta and Christini, 2012; Groenendaal et al., 2014; Colman, 2019).

### Plasticity of CRU Structure and Function in Health and Disease

Based on the above discussions, the impression may be that while CRU organization is very complex, it is at least static. However, a wealth of data indicate that, in fact, there is remarkable plasticity of dyadic structure and function. Even basic confocal imaging studies have long established that TT organization is highly malleable, as these structures appear gradually during development and are significantly reorganized during disease (reviewed in Setterberg et al., 2021). This structural reorganization leaves functionally “orphaned” RyRs, which are no longer present in dyads, and these sites exhibit delayed Ca^2+^ release during the action potential only after diffusion of trigger Ca^2+^ from intact dyads (Louch et al., 2006; Song et al., 2006). Thus, the overall pattern of Ca^2+^ release across the cell is desynchronized and slowed. More recently, high-resolution imaging studies have provided even greater detail in examining nanoscale TT structure in health and disease. Using SMLM, non-SMLM techniques such as STED microscopy, and EM, several studies have reported that there is considerable variation in t-tubule diameter and in the extent of folding of their luminal membranes (Wagner et al., 2012; Hong et al., 2014; Jayasinghe et al., 2014; Crossman et al., 2017; Frisk et al., 2021). Importantly, t-tubules dilate during heart failure, and the intricate folding structure is lost (Hong et al., 2014; Crossman et al., 2017; Frisk et al., 2021). While it is possible that t-tubule dilation may result in misalignment of LTCCs and RyRs (Jones et al., 2018), modeling studies have indicated that loss of inner membrane folding increases the rate of solute exchange between the t-tubule lumen and extracellular space, in a pro-arrhythmic manner (Hong et al., 2014).

Sarcoplasmic reticulum structure is also known to be malleable, as classic EM studies revealed that jSR geometry differentiates gradually in the developing heart with initial appearance of jSR at the cell surface, prior to the later introduction of cisternae at t-tubule sites (Franzini-Armstrong et al., 2005). As mentioned above, SBF-SEM imaging has provided fantastic 3D detail of SR structure (Figure 2C), and indicated that there is an overall loss of SR during heart failure, including local lesions where structure is markedly disrupted (Pinali et al., 2013). Coincident with changes in SR structure, are alterations in the nanoscale arrangements of RyRs during disease. Indeed, several studies have reported that RyR clusters are broken apart in heart failure (Kolstad et al., 2018; Sheard et al., 2019) and atrial fibrillation (Macquaide et al., 2015). We have, with the help of mathematical modeling, linked this “dispersion” of RyR clusters and CRUs to slowing of spark kinetics during heart failure, as Ca^2+^ release spreads between sequentially activated clusters (Louch et al., 2013; Kolstad et al., 2018), desynchronization of the Ca^2+^ transient, and slowing of cardiomyocyte contraction (Bøkenes et al., 2008). Interestingly, Macquaide et al. (2015) linked similar rearrangements of RyRs to the generation of pro-arrhythmic Ca^2+^ waves, enabled by an increased appearance of fragmented CRUs extending between z-lines. The drivers of this RyR reorganization are as yet unclear. However, even in the healthy heart Asghari et al. (2020) observed that RyR position and orientation are sensitive to phosphorylation status, Mg^2+^ concentrations, and the presence or absence of accessory proteins such as FKBP12.6. We anticipate that unraveling these mechanisms and their consequences for Ca^2+^ homeostasis in the normal and diseased cardiomyocyte will be an important topic of future experimental and modeling analyses.

### Addressing Remaining Experimental Limitations

An exciting time is upon us, as experimentalists and modelers now find themselves nearly aligned in terms of the spatial scale that can be investigated. However, there remain significant experimental hurdles in directly linking nanoscale CRU structure and function, and this in turn leaves computational scientists without definitive validation data for their models. Specifically, the super-resolution imaging techniques described above generally require fixed cardiomyocytes, which has precluded directly pairing Ca^2+^ recordings to cellular substructure. One recent study attempted this feat in a correlative manner, with sparks recorded first in live cells, prior to fixation, and subsequent super-resolution imaging of the same CRUs (Hurley et al., 2021). However, it should be noted that with complex protocols for fixation, labeling, and imaging, there is considerable risk for disrupting cell geometry and alignment between imaging modalities. Another option is to use super-resolution imaging in live cells expressing a photo-activated fluorophore affixed to a protein of interest. Recent work has employed this approach to examine LTCC positions together with “sparklets”; i.e., Ca^2+^-dependent fluorescence events corresponding to single channel openings (Ito et al., 2019). Similar approaches could be envisioned to allow pairing of RyR localization and Ca^2+^ spark measurements in the same, living cell. This would enable direct investigation of RyR cooperativity within and between clusters, and definition of CRUs based on function rather than assumptions based solely on knowledge of RyR positions.

While there have been impressive recent strides taken in the advancement of light microscopy, the above discussion has made clear that SMLM techniques like dSTORM and PALM cannot adequately resolve the arrangement of proteins within clusters. Indeed, these techniques routinely yield an xy resolution of 30–40 nm which, even in the case of a large protein like the RyR (width ∼27 nm), is sufficient only for defining an area occupied by the channel. For insight into intra-cluster, real-time RyR location, the increased resolution of a technique like DNA-PAINT would be needed (Figure 3D), but with suitability for live cell experiments. By definition, live cells cannot provide the immobile “docking” strands that DNA-PAINT requires, and DNA “imager” strands may be rapidly degraded and/or associate with cellular DNA or RNA. Thus, live-cell applications of DNA-PAINT have so far been very limited, and only employed for imaging molecules on cell surfaces (Strauss et al., 2018). A very recent study surmounted the latter challenge by designing “docker” and ‘imager’ probes with left-handed DNA (L-DNA) which do not hybridize with endogenous nucleic acids (Geertsema et al., 2021). Still, it seems clear that continued refinement of these techniques will be necessary to gain further experimental insight into dynamic changes in CRU structure and function at the length scale of single RyRs.

Although much of this review has focused on developments in improving the spatial scale of imaging, it should be noted that the temporal scale of measurements is also a critical experimental limitation. While events such as RyR openings are quite brief (∼5 ms, given best estimates of simultaneous changes in jSR and cytosolic [Ca^2+^]), the frame time for recording Ca^2+^ release events is limited to approximately 2 ms with even the latest cMOS cameras. Thus, recorded Ca^2+^ signals are near the limit of Nyquist discrimination for single channel openings, and generally represent a complex integration of releases from multiple RyRs. One approach to addressing this issue was developed by the Lipp group (Tian et al., 2017). Termed “CaCLEAN,” this technique removes the Ca^2+^ released in previous frames (and its expected diffusion) to reveal only the newly released Ca^2+^ signal. It is hoped that this type of approach, together with the further development of camera technology (sufficient signal:noise performance despite high frame rates), will shed further light into the true nature of RyR functional dynamics.

The above discussion has indicated that there remain substantial limits in attaining adequate spatial and temporal resolution to discern CRU structure and function based on experiments alone. Surely, this is good news for mathematical modelers, but will modelers be side-lined when technology eventually enables imaging of individual dyadic proteins and their Ca^2+^ signals in live cells in real time? This seems highly unlikely because protein organization is only one determinant of function. Post-translational modifications such as phosphorylation add yet another layer of complexity, and impose an additional level of difficulty in terms of imaging. Still, this integration of single protein regulation and structural dynamics is a very high priority for the field. If experimental and modeling frameworks can be devised to discriminate the contributions of each, it will open the door to being able to truly understand how each class of mechanism influences a broad range of disease phenotypes. This in turn would have important implications for effective therapeutic targeting of those diseases. Interestingly, emerging data indicate that phosphorylation of RyRs need not occur uniformly across clusters (Sheard et al., 2019), suggesting that channels at different positions in the CRU may have different Ca^2+^ sensitivities. However, modeling work to date has assumed the same Ca^2+^ sensitivity for all channels (“blanket phosphorylation”). We thus expect that including the regulation of individual channels in mathematical models will be essential for fully describing CRU channel behavior, even as imaging techniques continue to advance. Perhaps more importantly, the manner in which RyR localization and regulation contribute to whole-cell function will likely require an entirely different level of integration across the spatial scales of cardiac ECC—from CRU to cell. In the following section we deal specifically with the challenges to developing modeling approaches that may be capable of that level of integration.

## The Coming Challenges of Interdisciplinary Design in Studying Cardiac ECC—A Computational Scientist’s Perspective

### The Unique Challenges of Modeling the Biology of Cardiac ECC

As described above, the characteristics of unitary SR Ca^2+^ release events (sparks and spikes) exhibit a range of dynamic regimes, such as latched release and stochastic attrition, which depend critically on the number and proximity of RyR channels in a local CRU domain. These shifts in dynamics represent the direct influence of stochastic properties of individual channels on the local ensemble dynamics. Furthermore, as mentioned, modern imaging has made it abundantly clear that the number of RyRs present in many CRUs (from <4 to >200 colocalized channels) spans a range that crosses these transitions in ensemble dynamics. Finally, spatial gradients in [Ca^2+^] resulting from continuum diffusion in both the cytosol and SR also clearly contribute to determining those dynamic transitions. In combination, these characteristics enforce several basic requirements for accurately modeling cardiac CICR. Specifically, the scales covered by the model should allow for the stochastic operation of single RyRs to interact with realistic variability in the size and spatial organization of RyR clusters, as well as diffusive transport of Ca^2+^ in the cytosol and SR. As we discuss below, these requirements impose a substantial computational burden when simulating CICR among multiple CRUs up to subcellular scales. Thus, while the local studies of Stern (1992), Cannell and Soeller (1997), Soeller and Cannell (1997), Hake et al. (2012), and many others have already provided critical insights to single CRU function, the scale-up of these models to interrogate data arising from serial sectioning EM and other modern imaging methods (see Figure 4) will meet major technical challenges.

### The Ideal Model of Cardiac Myocyte Ca^2+^-Induced Ca^2+^ Release

To discuss these challenges in concrete terms it first helps to construct the hypothetical “ideal” model. Given the recent advances in serial sectioning EM and SMLM described above, it seems plausible to expect that existing or near-term datasets could yield: (1) the spatial distributions and geometric sizes of all jSR terminals in an average cardiac myocyte [via serial sectioning scanning electron microscopy (SS-SEM)], (2) the total volume and regional volume densities of the network SR in an average cardiac myocyte (via SS-SEM), and (3) the variation in RyR cluster sizes and their spatial distribution in a representative (perhaps 20 μm^3^) region of an average cardiac myocyte (via SMLM). In principle, these datasets should be collected in similar cells. That is, for example, from the same species, same cardiac chamber, or otherwise similar in terms of any variable known to impart systematic differences in membrane ultrastructure or RyR localization. From these datasets it will be possible to construct representative distributions of the quantities (1–3), as well as many others, e.g., the volumetric density of jSR terminals that are not coupled to t-tubules. Of course, the characteristics of the membrane and RyR cluster geometries will not be spatially correlated because they will be collected in different cells. However, given sufficient data, some form of registration between the distributions should allow for assigning reasonable cluster sizes to individual jSR geometries. This approximation could be made considerably more precise with correlative SMLM-EM datasets. Still, to continue with our thought experiment; presuming these essential data (1–3 above) are available, it is in principle possible to construct a model with full geometric detail of all jSR terminals incorporating realistic RyR cluster sizes and inter-cluster distances for an intact myocyte. It is another matter entirely to determine if such a model could be simulated with fully stochastic RyR dynamics and local diffusive coupling.

### Computational Requirements of the Ideal Model

The most costly dynamics for simulating such an expansive model of CICR will certainly occur in the ∼20,000 dyads of the average ventricular myocyte. To reliably solve the local reaction-diffusion dynamics in the dyadic space during a Ca^2+^ spark, a spatial resolution (mesh edge length) of 12–15 nm is typically required (Hake et al., 2012; Kolstad et al., 2018). Recent data suggest that the dyadic width may be even narrower (∼10 nm) than has been presumed in the past, and thus further refinement may be required (Rog-Zielinska et al., 2021; Setterberg et al., 2021). Presuming 12 nm is approximately correct, the volume of each mesh block would be approximately 1.7 × 10^–9^ pL. In the most conservative simulation strategy, involving a homogeneous finite volume integration, this would amount to some 1.7 × 10^10^ mesh blocks. The dynamics, particularly of the local RyR release current are also relatively stiff in the dyad, and require time discretization Δt ∼ 0.1 μs using operator splitting solution schemes, even with analytic solution for the current itself. This also ignores the cost of implementing local electrodiffusion (as opposed to conventional Fickian diffusion), which generally requires considerably finer temporal resolution. Of course, the scale of the problem can be reduced by employing finite element approaches involving larger mesh blocks (thus fewer computational nodes) in regions where the transporter gating dynamics are less stiff and gradients are less severe. Still, this problem represents a substantial computational effort. To date, very large cardiac simulations involving both classical and more recent electrodiffusion models (Tveito et al., 2017; Jæger et al., 2021a) have been performed at a similar scale (several billion computational nodes), albeit using powerful compute resources and for very brief simulation times (Jæger et al., 2021b). As such, approaching this computational problem is likely to require similarly substantial computational resources, thoroughly optimized numerical implementations, and well-designed simulations of relatively short duration.

### Approximating the Problem

**FIGURE 5:**
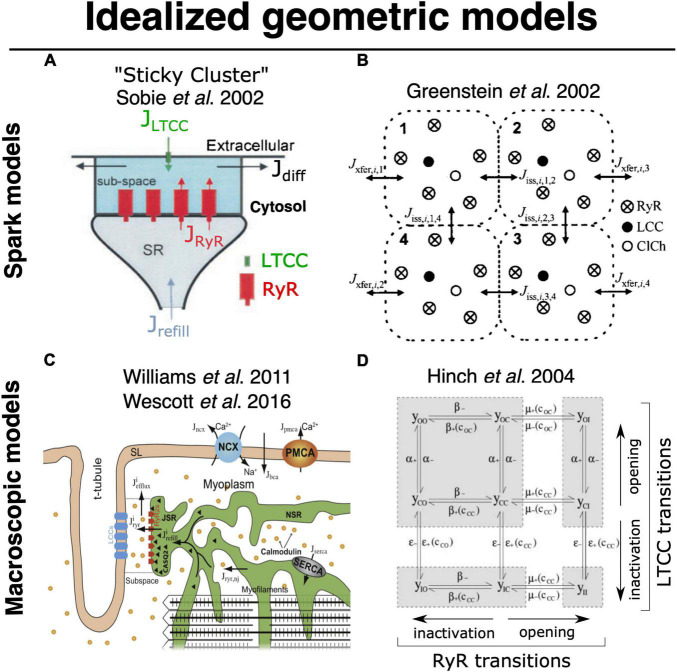
Foundational models for idealized geometric modeling of cardiac ECC. **(A)** Two important early dyadic structure models involving stochastic RyR dynamics were provided by Sobie’s sticky cluster (Sobie et al., 2002) **(A)**, and the 4-couplon model of Greenstein and Winslow (2002) **(B).** These two average dyadic models were each then extended to simulate whole-cell ECC by instantiating the 12,500–20,000 stochastically operating CRUs in the average ventricular myocyte Williams et al. (2011) and Wescott et al. (2016) **(C)**. Implemented models that solved stochastic RyR dynamics across these many identical sticky-cluster-derived CRUs, whereas Hinch et al. (2004) and Hinch (2004) performed a clever timescale decomposition to markedly reduce the scale of the problem **(D)**. Both families of model have been successful in replicating and/or interrogating a number of important macroscopic properties of ECC. However, neither is readily capable of assessing the impact of local RyR dispersion, and they do not implement known heterogeneities in CRU structure and function (see Section 2). Copyright permission to reproduce the figures.

The computational challenges imposed by simulating detailed stochastic dynamics across the comparatively large volume of a myocyte have been appreciated for some time. Some of the earliest efforts to perform this type of multiscale integration were begun by Ray Winslow’s group at Hopkins, and Robert Hinch at Oxford. Initially, Greenstein and Winslow (2002) implemented a whole myocyte with 12,500 CRUs, each with 4 coupled dyadic subspaces (to represent the intracellular compartment for each of 4 LTCCs) and a single local jSR (Figure 5B). These 12,500 CRUs were coupled to single compartments for the network SR and the cytosol. In 2002 this was a substantial computational effort even though it did not implement genuine continuum diffusion throughout these compartments. Hinch followed up by cleverly reducing the scale of the problem (Figure 5D; Hinch et al., 2004). He recognized that very rapid intradyadic Ca^2+^ diffusion implied that it was only the state of the channels (open or closed) that dictated the cleft [Ca^2+^] at any point in time. Thus, he was able to reduce the dyadic dynamics to a set of equations, the size of which was determined by the number of channels (both LTCCs and RyRs) assumed to be in each dyad. This in turn allowed grouping of the rate constants that applied at similar timescales to each channel species and permitted a 9-state (and even 4-state) model of each dyad. This strategy recapitulated the essential properties of local control, while markedly reducing computational cost. Unfortunately, this approach still assumes a single spatially homogeneous dyadic [Ca^2+^], which from the more recent super resolution studies described above seems unlikely to reflect the nanoscale reality.

More recent studies that have attempted to bridge the scales in a manner similar to what would be achievable via the ideal model (see previous section) have used a variety of simplifications to maintain tractability. Notably, the proof-of-principle work from Colman et al. (2017) described above used a range of approximations that prevented modulation of CRU size and avoided any potential for CRU fragmentation (i.e., dispersion of RyRs on the dyadic surface of the jSR), Figure 4B. Another range of computational studies from George Williams, Eric Sobie and Saleet Jafri has explicitly sought to simulate the whole-cell manifestations of dynamics that may be attributed to RyR dispersion in the single CRU studies described earlier (see Figure 5C) (Williams et al., 2011; Wescott et al., 2016; Sobie et al., 2017). Specifically, they investigated the role of changes in functional coupling between RyRs to explore the determinants of visible and “silent” SR Ca^2+^ leak (which represents sub-spark release events such as quarks) in a whole cell model involving distributed dyads. These clever studies diffusively coupled their 20,000 jSR terminals to a common network SR and employed thorough mass conservation and reuptake-release balance. Their dyadic model utilizes a modified version of the well-known “sticky-cluster” model developed by Sobie (Figure 5A; Sobie et al., 2002). This formulation implements an arbitrary number of RyRs at each dyad, each of which senses a common dyadic Ca^2+^ concentration, but for which an inter-RyR coupling term reflects the ability for opening and closing events in each RyR to trigger analogous events in other members of the cluster. By updating the original Ca^2+^ sensitivity of the RyR model, Wescott et al. (2016) were able to completely relax this coupling term and assess the effect of RyR mutations present in catecholaminergic polymorphic ventricular tachycardia (CPVT) on whole myocyte silent leak and ECC. In principle, through its inter-RyR coupling term, this model provides a phenomenological approach capable of recapitulating changes in inter-RyR coupling due either to dispersion or presumed inter-RyR interactions accompanying phosphorylation.

### Discriminating the Roles of RyR Regulation and Localization

The above approximations are important first steps toward simulating whole-myocyte CICR while retaining the critical stochastic characteristics of RyR gating. However, their ability to simultaneously discriminate regulatory effects (e.g., phosphorylation) that impact RyR Ca^2+^ sensitivity from changes in RyR cluster morphology or other disease-associated structural changes can only be described as phenomenological. To fully simulate the interaction of these changes, which are generally observed simultaneously in disease, will provide a key foundation for understanding whether individual changes in dyadic structure and single channel function contribute to the pathology, or if either is in fact compensatory in nature. As mentioned above, the ability to integrate these effects over an entire cell will allow us to begin these interrogations. This can be considered one of the major goals of the new modeling frameworks that can now be developed based on state-of-the art SSEM and SMLM imaging.

### Looking Forward to Image-Driven Multiscale Simulation of Cardiac ECC

In 1960, Wigner (1960) discussed the almost mystic efficiency of mathematics in physics; “the enormous usefulness of mathematics in the natural sciences is something bordering on the mysterious and there is no rational explanation for it.” Forty-five years later, Cohen (2004) expressed similar—or even stronger sentiments—regarding the application of mathematics in Biology. Cardiac physiology has a history rich with successful integration of experimentation and mathematics. The storied work of Hodgkin and Huxley (1952a,b,c, d) and Hodgkin et al. (1952) gave every reason for optimism, and the same methods were quickly applied to understand cardiac electrical activation (Noble, 1962). From these formalisms, continuum mathematical representations were developed to understand many aspects of macroscopic cardiac function (Plonsey and Barr, 1987; Henriquez, 1993, 2014; Henriquez et al., 2004; Roberts et al., 2008; Tveito et al., 2017; Jæger et al., 2021a). As many of the imaging methods described above have developed, it has become apparent that nanometer scale structures and their functional dynamics make critical contributions to cardiac electrophysiology and contractile function. Specifically in cardiac ECC, the value of mathematical methods in earlier years developed around their ability to address these nanoscale mechanisms. This type of modeling has been built upon studies of patch clamp electrophysiology and protein biochemistry, which have provided, and will continue to provide, our understanding of the functional building blocks of cardiac ECC. However, it is likely that mathematical approaches will become even more important for integrating experimental observations across the range of accessible scales. To achieve this will require development of efficient and automated handling of large SSEM and SMLM datasets, carefully designed heuristics to simulate CICR across at least 3 spatial orders of magnitude, and efficient but robust numerical approaches to solving the underlying systems of equations. These constitute major computational challenges, but appear to be within reach.

## Concluding Remarks

Over the history of cardiac ECC there have been many instances in which technological or methodological advances have permitted step-changes in the progression of knowledge and shifts in the working paradigm. Rarely, however, has there been a period in which a range of major and complementary technical developments have occurred in such quick succession, as has been the case over the past decade. Like many others, we are eager to see what these developments yield for the field. We particularly expect data sources that are now becoming available to provide a foundation for reconciling multiple levels of investigation and postulates from past decades. We hope this, in turn, will provide new tools and approaches for understanding the relationship between dynamic nanoscale changes and whole-cell function, and new insight to mechanisms of disease that span these scales. Perhaps more than any other aspect, we expect this period to require integrated design of experiments and simulations to fully leverage the power of these new technologies and methods.

## Author Contributions

WL and AE co-designed, wrote, edited, and developed figures for manuscript. HP-D wrote, edited, and developed figures for manuscript. All authors contributed to the article and approved the submitted version.

## Conflict of Interest

The authors declare that the research was conducted in the absence of any commercial or financial relationships that could be construed as a potential conflict of interest.

## Publisher’s Note

All claims expressed in this article are solely those of the authors and do not necessarily represent those of their affiliated organizations, or those of the publisher, the editors and the reviewers. Any product that may be evaluated in this article, or claim that may be made by its manufacturer, is not guaranteed or endorsed by the publisher.
